# Non-Communicable Diseases in Emergencies: A Call to Action

**DOI:** 10.1371/currents.dis.53e08b951d59ff913ab8b9bb51c4d0de

**Published:** 2013-09-06

**Authors:** Alessandro Demaio, Jennifer Jamieson, Rebecca Horn, Maximilian de Courten, Siri Tellier

**Affiliations:** Copenhagen School of Global Health, University of Copenhagen, Copenhagen, Denmark; The Alfred Hospital, Melbourne, Victoria, Australia; ChronAid, Washington, DC, USA; University of Copenhagen, Copenhagen, Denmark; Copenhagen School of Global Health, University of Copenhagen, Copenhagen, Denmark

## Abstract

Recent years have demonstrated the devastating health consequences of complex emergencies and natural disasters and thereby highlighted the importance of comprehensive and collaborative approaches to humanitarian responses and risk reduction. Simultaneously, noncommunicable diseases are now recognised as a real and growing threat to population health and development; a threat that is magnified by and during emergencies. Noncommunicable diseases, however, continue to receive little attention from humanitarian organisations in the acute phase of disaster and emergency response. This paper calls on all sectors to recognise and address the specific health challenges posed by noncommunicable diseases in emergencies and disaster situations. This publication aims to highlight the need for: • Increased research on morbidity and mortality patterns due to noncommunicable diseases during and following emergencies; • Raised awareness through greater advocacy for the issue and challenges of noncommunicable diseases during and following emergencies; • Incorporation of noncommunicable diseases into existing emergency-related policies, standards, and resources; • Development of technical guidelines on the clinical management of noncommunicable diseases in emergencies; • Greater integration and coordination in health service provision during and following emergencies; • Integrating noncommunicable diseases into practical and academic training of emergency workers and emergency-response coordinators.

## Background

In the 21^st^ century, we live in a world regularly affected by emergencies^[1]^, often with severe local and regional health consequences. Recent examples include the devastating hurricanes and floods in the US, Australia and Pakistan; earthquakes in Haiti, Japan, and New Zealand; and the conflict in Syria. In the context of climate change and corrosive political instability in many world regions, it is unlikely that we will see a reduction in disastfers^[2]^ or their resulting health impacts.

Simultaneously we are witnessing an increase in the burden of noncommunicable diseases (NCDs), including, but not limited to, heart disease, diabetes, cancers and chronic lung diseases. This phenomenon is not only visable in aging populations, but now too in younger populations secondary to the obesity epidemic and more sedentary lifestyles. Described by UN Secretary General Ban Ki-Moon as a “public health emergency”, NCDs are already the world’s leading cause of death. The diseases are estimated to be responsible for over 60% of global mortality each year with 80% of these deaths occurring in low- and middle-income settings^1^. In addition, NCDs now contribute 54% of global Disability-Adjusted Life Years Lost with a majority of this burden afflicting developing countries^2^.

NCDs also act as a barrier to economic development and equity causing financial and social stress^3^^,^ 4 ^,^ 5. In 2005, NCDs cost the Indian economy an estimated US$9 billion with an estimated 2 million people experiencing ‘catastrophic’ spending as a result of cardiovascular disease and cancer^6^.

While it is widely accepted that emergency situations render this vulnerable population increasingly susceptible to overcrowding, inadequate sanitation, poor shelter, insufficient food supply and disruptions to healthcare services^3^^,^ 7 ^,^ 8, few interventions are specifically aimed to mitigate these effects. These factors can also interact synergistically to result in an increased incidence of NCDs as well as progression of existing disease. In this light, NCDs are a poverty-cycle catalyst, exacerbated during a disaster.

During and following emergencies, there is often a fragmented approach to health intervention and coordination. Health authorities may not have the capacity to monitor and evaluate efforts and there is no single agency with the tools, resources and authority to take up this role^9^. Health systems can be compromised and health policy formulation disrupted^8^^,^ 10. Population health needs escalate and humanitarian personnel enter the arena to provide essential services^9^^,^ 11.

Research has been conducted and guidelines developed for the acute phase of disasters however these primarily focus on communicable diseases such as measles and diarrhoeal disease 12 . Limited research has been conducted into the short and long-term impacts and management of NCDs. There is a resulting paucity of NCDs in operational emergency guidelines and policies, for example:

- The Inter Agency Steering Committee (IASC) has no guidelines for NCDs, although there is a guideline on older persons that mentions the need for medication for chronic diseases;
- The Inter Agency Emergency Health Kit, coordinated by the WHO, contains limited medications for NCDs (e.g. no insulin in the current version);^14^
- Although the WHO has invested in producing the Field Manual – Communicable Disease Control in Emergencies, it has not produced a similar resource on NCDs^12^ - though the WHO will soon release new clinical guidelines on effective mental health care for adults and children exposed to trauma and loss;28
- The 2012 UK Government report "Reducing Risks of Future Disasters" importantly mentions the "mental health damage" associated with disasters as well as disability resulting from trauma, but provides no explicit direction on disaster risk reduction with regards to concomitant chronic disease burden, or resulting morbidity associated with other NCDs; 27
- The most recent edition of the Sphere Guidelines includes 40 pages on health, but only one of those paragraph refers to NCDs.^13^

The outcome is greater morbidity resulting from a lack of evidence-based guidelines and a resulting healthcare gap for populations with chronic diseases during and following emergencies. This gap, and the resulting morbidity, is yet to be sufficiently quantified.

## The Result of Noncommunicable Diseases in Emergencies

When considering NCDs and emergencies, the focus should be on the overall goals of the health response, particularly in the acute phases of an emergency. Effective emergency action can avoid the escalation of an event into a disaster. Emergency management involves plans and institutional arrangements to engage and guide the efforts of government, non-government, voluntary and private agencies in comprehensive and coordinated ways to respond to the entire spectrum of emergency needs. Its goal is directed towards avoiding excess morbidity and mortality (UNISDR).

There are several ways in which excess morbidity and mortality related to NCDs during emergencies and disasters might occur, for example:

**a)** **Persons with NCDs are more vulnerable in emergencies and disasters**

For individuals with NCDs, their condition may deteriorate as a direct result of the emergency. They are less able to cope without access to adequate nutrition, medications and follow-up^7^^,^ 11 ^,^ 15. This includes individuals living with disabilities. For example, people with diabetes may loose glucometers and insulin stock, lacking essential back-up supplies^8^. Dialysis centres may be destroyed and home healthcare services may not be operational^10^.

**b)** **Emergencies exacerbate NCDs leading to acute complications**

Many NCDs require close and sustained interaction with health systems and providers. During and following an emergency, this is often not possible. Due to interruptions in access to care and medications, acute exacerbations of NCDs can occur^16^^,^ 17. Common examples include exacerbations of chronic respiratory diseases and infectious ulcers in persons with diabetes mellitus^8^. Conditions that caused little or no impact on activities of daily living may deteriorate causing not only greater morbidity through debilitating symptoms but also loss of income, security^8^^,^ 10, limb or life.

**c)** **Long-term implications of NCDs resulting from emergencies and their management**

The morbidity and disability associated with NCDs is normally life-long. Therefore, suboptimal management during and after a disaster not only has immediate health effects, but can also have lasting social and health ramifications. A lack of appropriate care for even a short period can result in greater levels of chronic morbidity and suffering, as well as poverty entrenchment^3^.

**d) The multifaceted impact of NCDs and emergencies**

Developing countries are often disproportionately burdened by both NCDs and disasters in comparison to higher income countries^18^. Developing countries face the greatest burden from global and regional conflict as well as increased vulnerability to the effects of climate change and natural disasters. Many of these situations are exacerbated by the increasing levels of urbanisation and slum-populations. Additionally, the "double burden"^19^ of disease also contributes to the multifaceted impact of NCDs and emergencies.

## NCDs in Emergencies and Disasters: A Way Forward

In order to address the problem of NCDs in emergencies and minimise excess morbidity and mortality, the authors of this paper suggest:

**1.** **Comprehensive review of the current scientific literature**

A full and comprehensive review of the current global scientific literature relating to NCDs in emergencies and disaster situations is needed in order to build evidence-based policies and guidelines. Existing research should be evaluated, allowing for assessment of current interventions and identification of future interventional possibilities. In recent years, there have been several efforts to try and coordinate information, needs assessments, and disaster response, such as the efforts by the Cochrane review with EvidenceAid. Although admirable, these efforts continue to exclude specific focus on management of chronic diseases in disaster response at present.

Uniquely, a full assessment of evidenced-based practice in assessing and treating chronic disease in disaster may not be plausible in the classical sense since this is more of a "modern era epidemic." In decades past, chronic diseases did not affect the sheer number of people that they do now, nor did they present as a pressing consideration during a disaster. As a result, it will be vastly important to also collect and assess anecdotal evidence from first responders, humanitarian organisations, and governements who have recently experienced a large scale disaster to be able to fully understand the scope of chronic diseases in disaster and emergency settings.

**2.** **Increased awareness of the importance of NCDs in emergencies**

Greater advocacy and awareness surrounding NCDs and emergencies is vital. Discussion of NCDs specific to emergency response at the global level is imperative, as well as appropriate allocation of funding specific to chronic disease in disaster. Strides towards this have indeed been underway in venues such as the Geneva Health Forum, the American Public Health Association, as well as organization specific fora such as the Red Cross Movement and Medecins sans frontieres. Over the last couple of years the authors of this article have involved themselves in all of the above, supporting the discussions.

**3. Increased research on morbidity and mortality patterns during and following an emergency**

Further research is needed with regard to the patterns of morbidity and mortality related to NCDs in emergencies. Acute complications, long-term complications and disease progression need to be qualified and quantified. Health outcome assessments of disasters should incorporate NCDs as a factor impacting the current and potential health problems in a population affected by a disaster along the proposals made during the 15th World Congress on Disaster and Emergency Medicine in Amsterdam (2007) regarding assessing the public health dimension of disasters.

One viable option would be the establishment of a coordinated, international, open database focusing on the epidemiology of NCDs during and following emergencies around the world. A platform for government, academic, NGO and IGO data, this could serve as a timely, efficient and effective source of valuable evidence for policy and practice^26^.

**4. Incorporation of NCDs into existing emergency-related policies, standards, and resources**

NCDs should be incorporated into publications and operational guidelines and resources including the Sphere Handbook 13 and the Interagency Emergency Health Kit 14 ^,^ 20 ^,^ 21 . These must be readily available to those working within emergency situations.

Disaster risk reduction plans aiming to avoid, lessen or transfer the adverse effects of hazards through activities and measures for prevention, mitigation and preparedness (UNISDR) should assess and integrate the role of NCDs as a factor increasing the vulnerability of the population exposed. In this regard, the United Nations-endorsed Hyogo Framework for Action and the International Strategy for Disaster Reduction (ISDR) should recognize NCDs as *a* threat to achieve their expected outcomes to reduce losses related to disasters.

**5. Development of technical guidelines on the clinical management of NCDs in emergencies and disaster situations**

Guidelines, which take into account the practical problems associated with NCDs in emergencies are essential^21^. These could include partnerships and protocols for the supply of medications such as established by the International Diabetes Federation on insulin supply during emergencies and disasters (http://www.idf.org/insulin-diabetes-supplies/emergencies-and-disasters).

**6. Greater integration and coordination in health service provision during and following emergencies**

The integration of NCDs into emergency healthcare provision during and following emergencies is imperative. Collaboration between existing health infrastructure, the healthcare system and humanitarian assistance would improve the sustainability of efforts^22^. This does not require the duplication or reinventing of response efforts. Rather, an intelligent and collaborative approach learning from experiences relating to communicable diseases^23^. For example, health clinics and supply chains created for the treatment of malaria or diarrhoeal diseases should be capitalised in their potential to simultaneously avert excess suffering and disease from NCDs such as diabetes^24^ .

**7. Integrating NCDs into practical and academic training of emergency workers and emergency-response coordinators**

High-quality courses are required in order to build community-level, government and organisational capacity in the field of NCDs during and following emergencies and for disaster risk reduction planning. These courses should be practical, evidence-based and affordable to participants from every socio-economic background. Here again, several of the authors have been involved in developing such training.

## Our Call to Action

We call upon healthcare professionals, communities, organisations and governments to further understand and address the structural determinants of NCDs in emergency and disaster situations.

We urge governments, non-government organisations and intergovernmental bodies to allocate specific resources for the prevention and management of excess morbidity and mortality from NCDs in emergencies and disasters on a scale which reflects the magnitude of this health issue.

We call on the international research, humanitarian and governing sectors to recognise and address NCDs in emergency and disaster situations and produce evidence-based, global technical guidelines for the management of NCDs in emergencies and disasters. NCDs should not have a token inclusion, but rather a meaningful and integrated one that addresses the care gap for this vulnerable population.

## Footnote

^[1]^For the purposes on this publication, emergencies include natural disasters, conflicts and technological disasters, or a combination, which result in major loss of life in a population and disruption to healthcare.

^[2]^In this publication, disaster refers to a serious disruption of the functioning of a community or a society involving widespread human, material, economic or environmental losses and impacts, which exceeds the ability of the affected community or society to cope using its own resources. Disasters are often described as a result of the combination of: the exposure to a hazard; the conditions of vulnerability that are present; and insufficient capacity or measures to reduce or cope with the potential negative consequences. Disaster impacts may include loss of life, injury, disease and other negative effects on human physical, mental and social well-being, together with damage to property, destruction of assets, loss of services, social and economic disruption and environmental degradation”^25^.

## Competing Interests

The authors of this paper declare no conflicts of interest.
